# Integrative analysis of Iso-Seq and RNA-seq data reveals transcriptome complexity and differential isoform in skin tissues of different hair length Yak

**DOI:** 10.1186/s12864-024-10345-8

**Published:** 2024-05-21

**Authors:** Xuelan Zhou, Xiaoyun Wu, Chengfang Pei, Meilan He, Min Chu, Xian Guo, Chunnian Liang, Pengjia Bao, Ping Yan

**Affiliations:** 1 Key Laboratory of Animal Genetics and Breeding on Tibetan Plateau, Ministry of Agriculture and Rural Affairs, 730050 Lanzhou, P.R. China; 2 Key Laboratory of Yak Breeding in Gansu Province, Lanzhou Institute of Husbandry and Pharmaceutical Sciences, Chinese Academy of Agricultural Sciences, 730050 Lanzhou, P.R. China; 3 Animal Husbandry Technology Promotion Station of Tianzhu County, 733000 Wuwei, P.R. China

**Keywords:** Tianzhu white yak, Hair length, Isoform sequencing, RNA-seq, Differential isoforms

## Abstract

**Background:**

The hair follicle development process is regulated by sophisticated genes and signaling networks, and the hair grows from the hair follicle. The Tianzhu white yak population exhibits differences in hair length, especially on the forehead and shoulder region. However, the genetic mechanism is still unclear. Isoform sequencing (Iso-seq) technology with advantages in long reads sequencing. Hence, we combined the Iso-seq and RNA-seq methods to investigate the transcript complexity and difference between long-haired yak (LHY) and normal-haired yak (NHY).

**Results:**

The hair length measurement result showed a significant difference between LHY and NHY on the forehead and the shoulder (*P-value* < 0.001). The skin samples from the forehead and the shoulder of LHY and NHY were pooled for isoform sequencing (Iso-seq). We obtained numerous long transcripts, including novel isoforms, long non-coding RNA, alternative splicing events, and alternative polyadenylation events. Combined with RNA-seq data, we performed differential isoforms (DEIs) analysis between LHY and NHY. We found that some hair follicle and skin development-related DEIs, like *BMP4*, *KRT2*, *IGF2R*, and *COL1A2* in the forehead skin; *BMP1*, *KRT1*, *FGF5*, *COL2A1*, and *IGFBP5* in the shoulder skin. Enrichment analysis revealed that DEIs in both two comparable groups significantly participated in skin and hair follicle development-related pathways, like ECM-receptor interaction, focal adhesion, and PI3K-Akt signaling pathways. The results indicated that the hair follicle development of Tianzhu white yak may influence the hair length difference. Besides, the protein-protein interaction (PPI) network of DEIs showed *COL2A1* and *COL3A1* exhibited a high degree of centrality, and these two genes were suggested as potential candidates for the hair length growth of Tianzhu white yak.

**Conclusions:**

The results provided a comprehensive analysis of the transcriptome complexity and identified differential transcripts that enhance our understanding of the molecular mechanisms underlying the variation in hair length growth in Tianzhu white yak.

**Supplementary Information:**

The online version contains supplementary material available at 10.1186/s12864-024-10345-8.

## Background

The hair coat of domestic animals serves essential functions in their environmental adaptation. It helps animals regulate their body temperature and protects against insect bites. Additionally, certain domestic animals, such as wool sheep, angora rabbits, and yak, are utilized for textile materials [1]. The wool production of animals holds significant economic value in the development of animal husbandry and the textile industry [2]. Yak (*Bos grunniens*) is a bovine species, and most yaks live in the Qinghai-Tibet Plateau of China. As domestic livestock, yaks are well adapted to the high-altitude environment and produce meat, milk, and other resources for human use. The Tianzhu white yak is a Chinese indigenous yak breed that could be easily distinguished from other yak breeds due to their pure white hair coat. The well-proportioned coarse and fine hairs on their surface play a crucial role in helping yaks thrive in the harsh environment of high altitudes, including strong wind, low temperature, and intense solar radiation [3]. In addition, the growing demand for natural and ethically sourced materials in the textile industry has contributed to the increased value of yak hair. In the textile industry, white yak hair is more valuable than black yak hair due to white hair can be easily dyed with colors. It is worth noting that there is significant variation in hair length within the Tianzhu white yak population. It is worth noting that there is variation in hair length within the yak population. Previous research has indicated that the number, length, and diameter of hair can impact the environmental adaptability and wool quality of domestic animals [4–7]. Hair growth is a complex process and is intricately regulated by numerous genes and signaling pathways.

Fibroblast growth factor 5 (FGF5) has been suggested to be associated with hair length in various species [8–11]. Bao used the resequencing method to investigate the hair length-related genes and also identified *FGF5* as a candidate gene [12]. However, the molecular mechanism underlying the differences in hair length growth in yaks remains unclear. Transcription is the main process of DNA transfer information to protein, and the number and the expression level of transcripts could respond to the different biological processes in the organism. The RNA-seq method has been used to profile the transcriptome in many species, but, its short-read limitations make it less ideal for identifying individual gene isoforms. With the advancement of PacBio sequencing technology, the Single Molecule Real-Time (SMRT) technology could directly produce long-length reads [13]. Full-length transcriptome analysis provides valuable insights into transcriptomes, including gene isoforms, long non-coding RNA (lncRNA), alternative splicing (AS), and alternative polyadenylation (APA) events. Previous research has also suggested that post-transcriptional modification events could modulate gene expression in various biological processes [14]. For example, lncRNA has been shown to participate in the hair follicle cycle of yaks [15]. Alternative splicing of FGF5 produced a short FGF5 isoform, which prolonged the anagen VI phase of the hair follicle cycle by suppressing the activation of FGF5 at anagen and caused rats with long hair phenotype [16].

In the current study, we observed significant differences in hair length between long-hair yaks (LHY) and normal-hair yaks (NHY) when comparing the hair length on the forehead and shoulder. However, the specific genes responsible for controlling hair growth and its characteristics in yaks remain unknown. To gain a better understanding of the transcriptional profile of yak skin tissue between LHY and NHY. We used PacBio isoform sequencing (Iso-seq) technology to identify the post-transcriptional modification events in yak skin tissues and combined RNA-seq to investigate the differential isoforms (DEIs) in the skin tissue of the forehead or shoulder between LHY and NHY. This study aims to enhance our comprehension of the yak skin tissue transcriptome and aid in unraveling the genetic mechanisms underlying the diversity in yak hair length.

## Results

### Hair length trait analysis

**Fig. 1:**
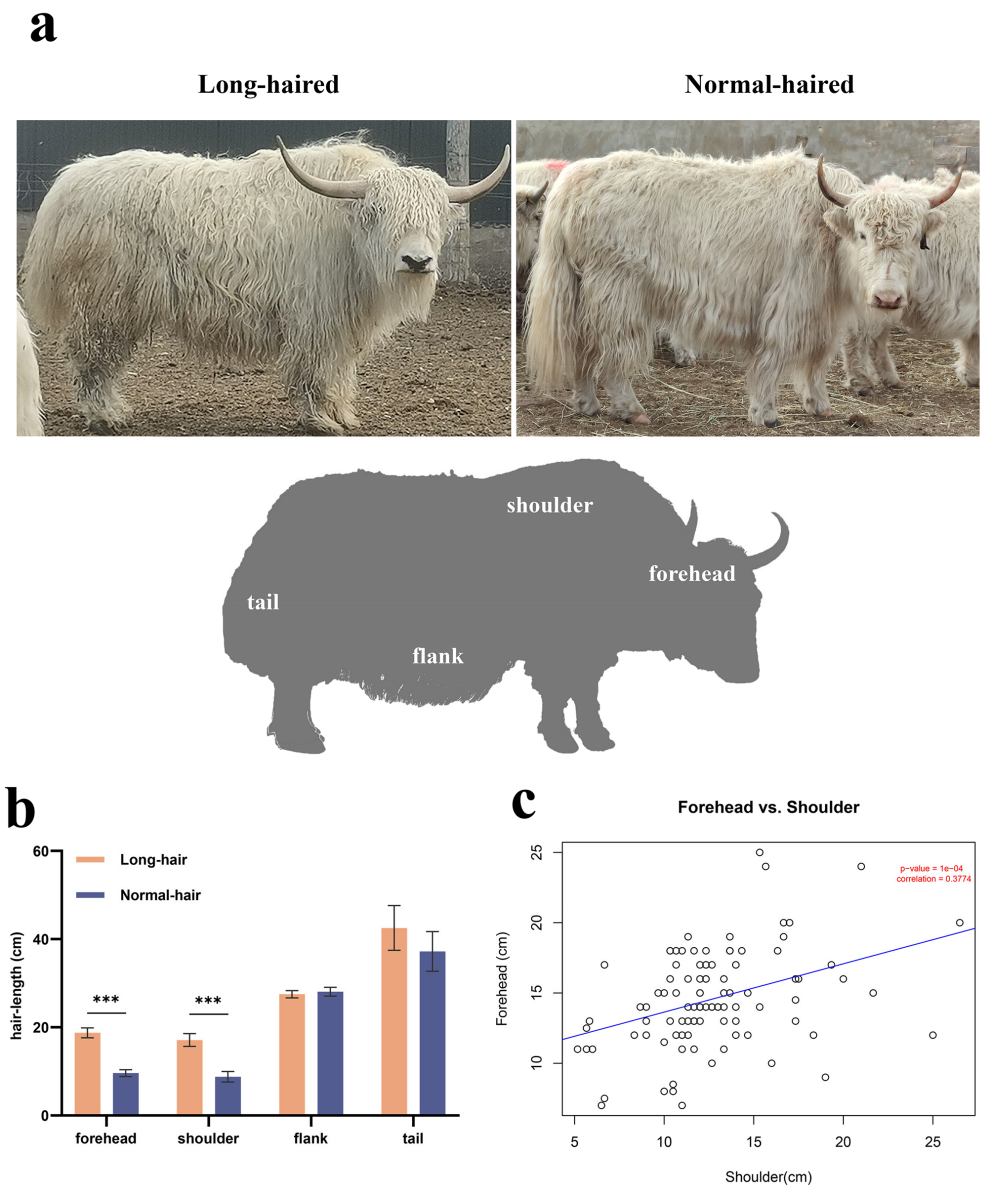
Statistical analysis of hair length in the Tianzhu white yak. (**a**) The appearance of the long-haired yak (left) and normal-haired yak (right). (**b**) The statistical difference of hair length in different body positions between LHY and NHY; (**b**) The correlation analysis of hair length between forehead and shoulder in the Tianzhu white yak

A total of 10 LHYs and 9 NHYs were used for hair-length measurement. The appearance of the yak and the body position of hair length measurement are shown in Fig. 1a. The statistical result showed the mean hair length of LHY is longer than NHY on the forehead, shoulder, and tail. Detailed data on hair-length measurement is supplied in Additional file 1. Notably, the mean hair length on the forehead and shoulder of LHY is approximately twice that of NHY. The t-test result showed the hair length on the forehead and shoulder with a significant difference between LHY and NHY (*P-value* < 0.001) (Fig. 1b). Pearson correlation coefficient analysis showed there was a significant correlation of hair length between the forehead and shoulder in Tianzhu white yak (R^2^ = 0.3774, *P-value* < 0.001) (Fig. 1c).

### Data summary of iso-seq

**Fig. 2:**
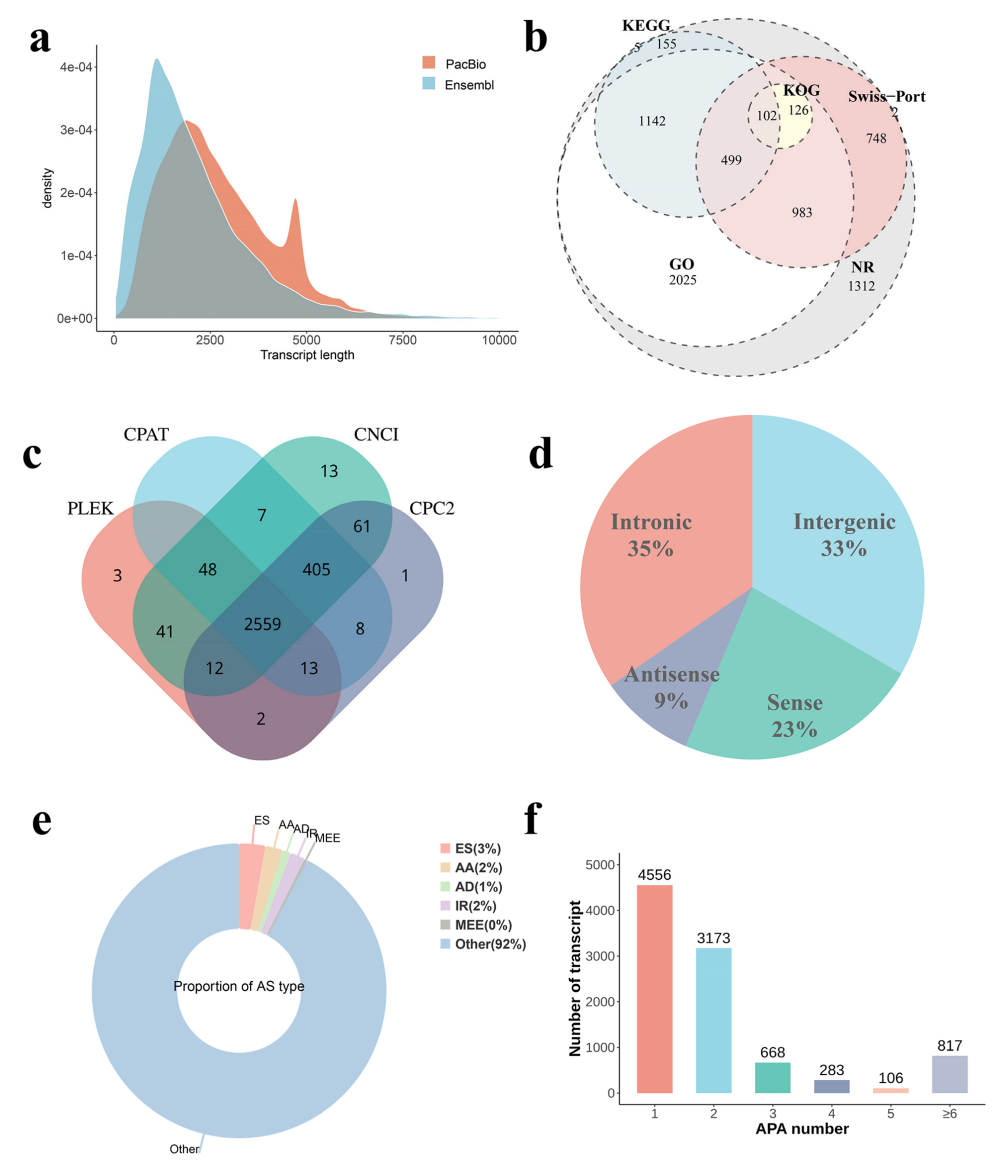
Date summary of Iso-seq. (**a**) The density of transcript length; (**b**) The Venn diagram of novel isoforms of novel genes; (**c**) The Venn diagram of lncRNA prediction; (**d**) The percentage pie of lncRNA classification; (**e**) The percentage pie of AS type; (**f**) Transcript statistics of different APA

PacBio long-reads, combined with next-generation RNA sequencing, were utilized to comprehensively identify the global transcripts in the skins of LHY and NHY. Iso-seq generated 51,90,023 polymerase reads. After filtering, we obtained 653,994 FLNC reads with a ploy A tail (Additional file 2). Subsequently, high-quality short-reads were employed to correct the sequencing error generated by the Iso-seq, resulting in an increased number of FLNC reads with a high PID value compared to pre-correction. The reference mapping ratio also improved, with 89.17% of FLNC reads mapped to the reference with a high PID value (Additional file 3). Transcript information from corrected FLNC reads was then identified based on the genome reference annotation file. Compared to the reference annotation, PacBio sequencing captured more long transcripts (Fig. 2a). Statistical analysis of loci length revealed that 23.57% of loci annotated by the PacBio ranged from 2-3Kb, while 41.19% of loci were greater than 3Kb, indicating PacBio technology’s capability to sequence longer loci. Moreover, PacBio annotation identified more isoforms than ensemble annotation. (Additional file 4). The new annotation file used in this study was merged with the reference annotation and PacBio annotation (Additional file 5).

### Functional annotation result of novel isoform and LncRNA prediction

To understand the potential function of the identified novel isoforms, their sequences were blasted to multiple public databases for functional annotation. The analysis revealed that 9,845 novel isoforms of novel genes were annotated in NR (7,285, 74.00%), GO (4,877, 49.54%), KO (5,316, 54.00%), KOG (250, 2.54%), and Swiss-Prot (2,653, 26.95%) databases (Fig. 2b). Full-length transcript sequencing is a useful strategy for lncRNA identification. All the novel isoforms of annotated and novel genes were predicted for their protein-coding potential using the NR, KEGG, KOG, and Swiss-Port database. Then, the remaining novel isoforms were used for the lncRNA prediction using CPC2, CPAT, PLEK, and CNCI software. Finally, 2,559 lncRNA were identified as reliable predictions, as they were simultaneously predicted by all four methods (Fig. 2c). The genome location result showed the predicted lncRNA was generated from Intergenic (854, 33.37%), sense (588, 22.98%), antisense (230, 8.99%), and Intronic (887, 34.66%) (Fig. 2d).

### AS and APA events

In the present study, the AS event was identified. In total, 239,734 AS events were identified, including exon skipping (6,886, 2.87%), alternative acceptor (4,570, 1.91%), alternative donor (2,292, 0.96%), intron retention (3,893, 1.62%), mutually exclusive exon (783, 0.33%) and others (221,310, 92.31%) (Fig. 2e). The APA event could influence gene expression through miRNA regulation. In this study, a total of 12,214 APA sites of 7,313 genes were identified by Tapis software. There are 2,760 genes with 2 or more APA sites (Fig. 2f). There were also some keratin-associated genes with two or more APA sites. Especially, the result revealed that *ENSBGRG00000003029*, *CLI4*, *COL1A2*, *CANX*, and *THRSP* with more than 10 APA sites (Additional file 6).

### Functional enrichment analysis of the DEIs between NH and LH group

**Fig. 3:**
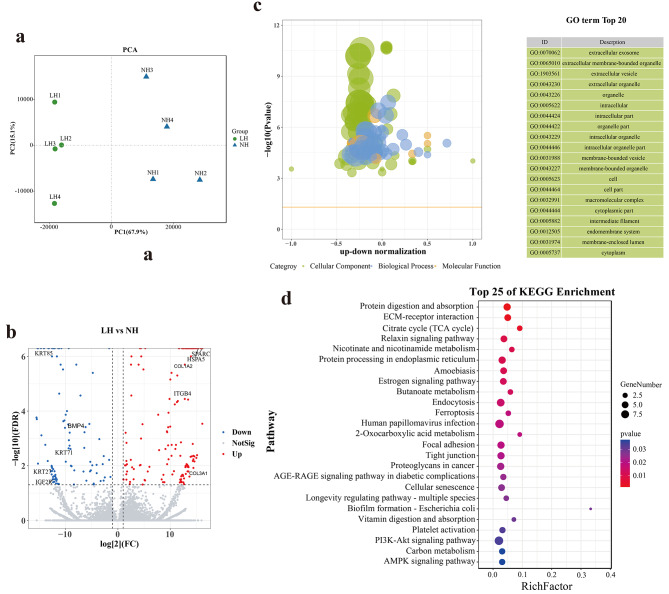
The DEIs analysis of forehead skin between LHY and NHY. (**a**) PCA analysis of 8 forehead skin samples; (**b**) Volcano plot of DEIs; (**c**) Top 20 GO terms of DEIs (*P-value* < 0.05); (**d**) Top 25 KEGG pathways of DEIs (*P-value* < 0.05)

The PCA analyses of forehead comparable groups showed that the samples between LH and NH groups could be distinct two parts according to PCA1, and the PCA1 value accounts for 67.9%. (Fig. 3a). Differential analysis between LHY and NHY revealed that 262 isoforms of 233 genes were differently expressed in the forehead skin, including 165 up-expressed isoforms in LH group and 97 up-expressed isoforms in NH group (Fig. 3b). GO enrichment result of DEIs be-tween forehead comparable groups were significantly enriched in extracellular exosome, extracellular membrane-bounded organelle, and extracellular vesicle terms (Fig. 3c). KEGG enrichment result of DEIs between forehead comparable groups significantly participated in some hair growth-related signaling pathways, such as ECM-receptor interaction, focal adhesion, and PI3K-Akt signaling pathway (Fig. 3d).

### Functional enrichment analysis of the DEIs between LS and NS group

**Fig. 4:**
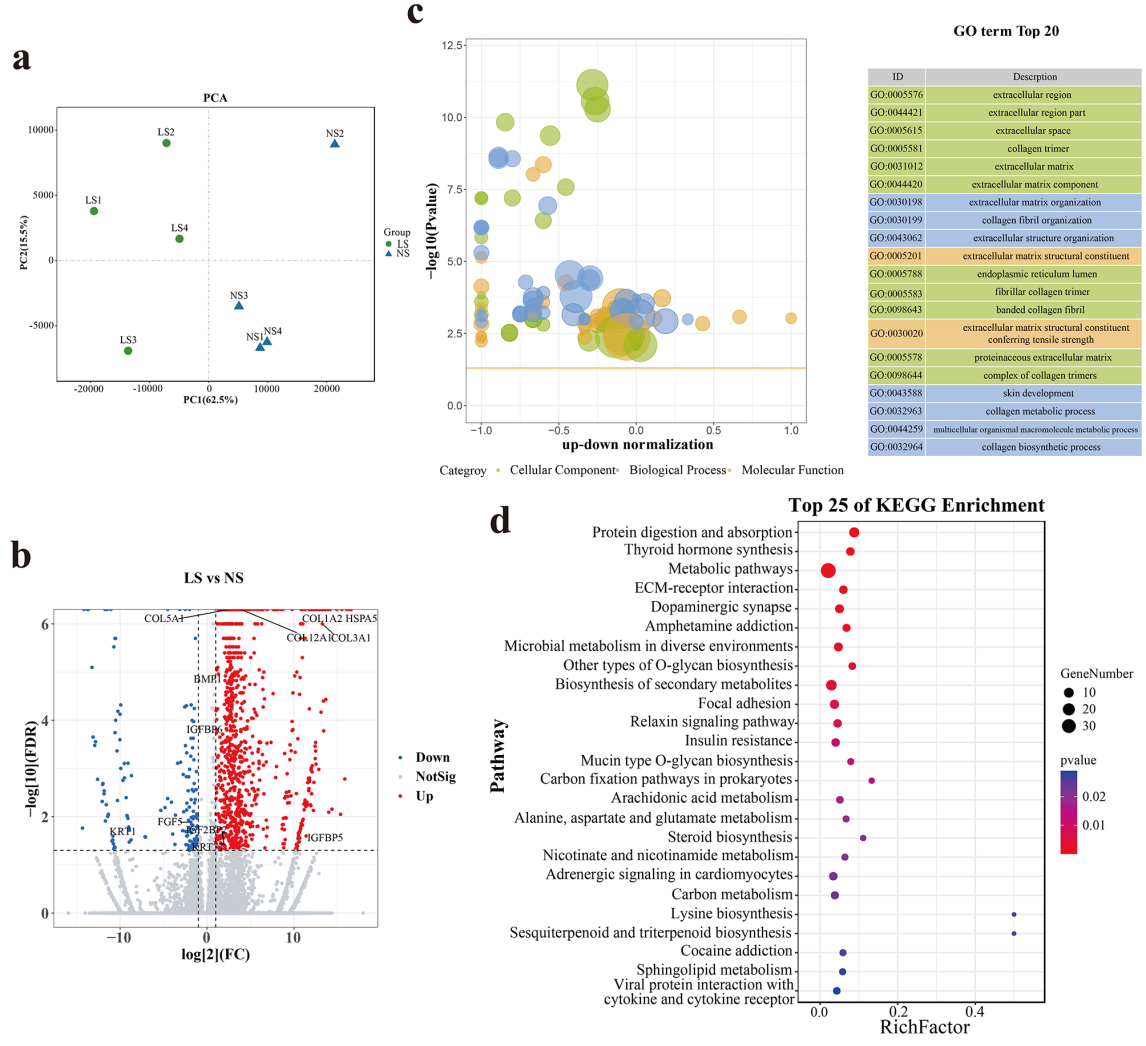
The DEIs analysis of shoulder skin between LHY and NHY. (**a**) PCA analysis of 8 shoulder skin samples; (**b**) Volcano plot of DEIs; (**c**) Top 20 GO terms of DEIs (*P-value* < 0.05); (**d**) Top 25 KEGG pathways of DEIs (*P-value* < 0.05)

According to the PCA analyses, the samples among shoulder comparable groups could be distinct two clusters depending on the PCA1, and the PCA1 contributes to 62.5%. (Fig. 4a). A total of 1,763 isoforms of 293 genes were differently expressed between the NS and LS comparable groups, including 1,610 up-expressed isoforms in the NS group and 154 up-expressed isoforms in LS group (Fig. 4b). GO enrichment result of DEIs between the shoulder comparable group were significantly enriched in the extracellular region, extracellular matrix organization, and skin development terms (Fig. 4c). KEGG enrichment result of DEIs between NS and LS group significantly participated in protein digestion and absorption, ECM-receptor interaction, focal adhesion etc. (Fig. 4d).

### Protein-protein interaction network of DEIs

**Fig. 5:**
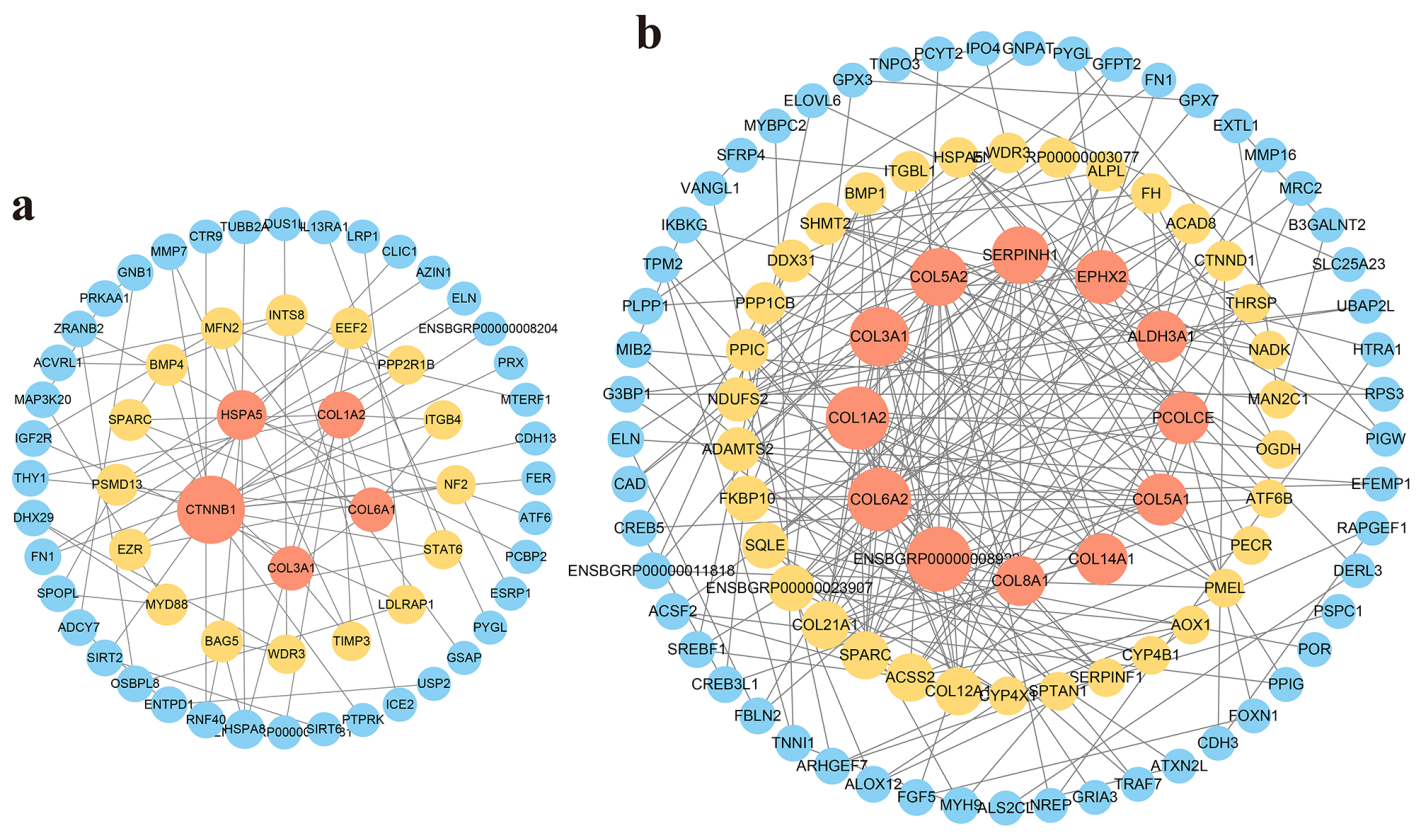
The PPI network of DEIs coding proteins, the size indicates node degree; (**a**) PPI network of DEIs in forehead comparable groups; (**b**)PPI network of DEIs in shoulder comparable groups

To further investigate the relationship between DEIs, the PPI analyses of DE-Is-associated proteins were performed. According to the PPI network, we found *CTNNB1*, *HSPA5*, *COL1A2*, and *COL3A1* proteins presented a high degree in the forehead comparable group (Fig. 5a). And the *ENSBGRP00000008923*, *SERPINH1*, *EPHX2*, *ALDH3A1*, *PCOLCE*, and some collagen family proteins like *COL6A2*, *COL1A2*, *COL3A1*, *COL5A2*, *COL5A1*, *COL14A1* with high degree in the shoulder comparable group (Fig. 5b). We found that *COL1A2* and *COL3A1* were both with high a degree in two comparable groups. We speculate that these two genes may play an essential role in hair length growth in the forehead and shoulder skin.

### Validation results

**Fig. 6:**
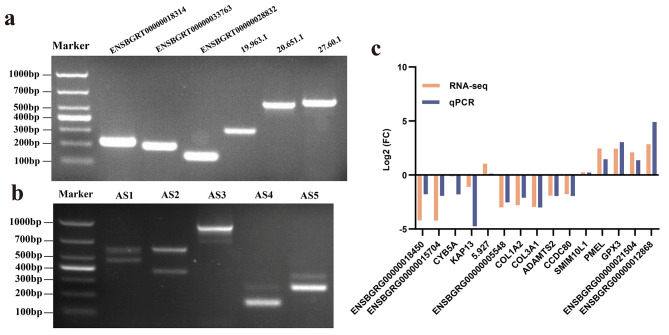
The validation result of Iso-seq and RNA-seq; (**a**) The PCR product length validation of known and novel isoform, the target gel was cropped from one full-length gel map rather than spliced; (**b**) The PCR product length validation of AS events, the target gel was cropped from one full-length gel map rather than spliced; (**c**) The tendency analysis of RNA-seq between RNA-seq and qPCR validation

To validate the reliability of Iso-seq and RNA-seq results, the isoform amplification and isoform relative expression analysis experiments were performed. The PCR and gel electrophoresis results showed the product length of 6 known and novel isoforms, and five AS events are consistent with the Iso-seq prediction results shown in the cropped gel picture (Fig. 6a, b), the original full-length gel figures are respectively supplied in the Additional file 7 and Additional file 8. Furthermore, the qPCR results also showed that the expression tendency of 15 selected isoforms is consistent with the RNA-seq result (Fig. 6c). The above validation results further indicated the reliability of the sequencing results in this study.

## Discussion

Hair coat serves multiple functions in animals, except for physical protection and body temperature regulation. Previous studies indicated that hair length correlates with milk production in cows and the ability of donkeys and mules to adapt to climate changes [17, 18]. Variations in hair length phenotype have also been observed in the Tianzhu white yak population. Previous research found evidence that hair length difference is related to the hair follicle cycle, for example, the long-haired rabbits with a long anagen phase of the hair follicle cycle [19]. In mammals, the periodic growth of hair follicles is controlled by a complex gene regulation network. The transcriptome study is a useful method to evaluate global gene expression and explore the key genes that were trait-related. PacBio sequencing with advantages in long transcript capture, lncRNA prediction, and AS and APA site identification [20]. Recently, numerous studies combined Iso-seq and RNA-seq to elucidate the transcriptome profiling in multiple species, like pigs [21], sheep [22], and red swamp crayfish [23]. To explore the genetic mechanism of hair length difference growth of yak, we integrated Iso-seq and RNA-seq to reveal the transcriptome complexity in yak skin and investigate the DEIs between LHY and NHY. In this study, a total of 9,845 novel genes and 66,989 isoforms were identified by PacBio seq, and nearly half of the gene loci were equal to or greater than 3Kb. Consistently with previous studies, PacBio identified more long transcripts than reference annotated transcripts [24, 25]. In addition, numerous novel lncRNA was predicted from the Iso-seq. The result confirmed the full length of the FLNC reads sequenced by the PacBio.

The post-transcriptional modification and regulation process can influence gene diversity and regulate gene expression. Previous studies have shown that the long hair phenotype in rats is caused by the loss of exon 2 of FGF5 [26]. In our study, a novel DEI of the *FGF5* gene was identified by the Iso-seq method. Additionally, we found the novel isoform with an ES event, and the splicing sites of the second exon is consistent with the known transcripts Non-coding RNAs have been reported with important roles in hair growth process regulation, such as a novel lncRNA-000133 from the secondary hair follicle may contribute to inductive property of dermal papilla cells in cashmere goat [27], and lncRNA H19 could maintain the hair inducing ability of dermal papilla cells [28]. Zhang analyzed lncRNA expression profiles during the hair follicle cycle of Tianzhu white yak and provided insights into the lncRNA regulation mechanism in the hair cycle [15]. In our study, we identified a total of 2,559 novel lncRNA using Iso-seq, some of which showed differential expression between LHY and NHY. All evidence suggested that PacBio sequencing is a useful method for the lncRNA study of hair follicle growth in the yak. In addition, miRNA was also studied with regulation function in hair growth. For example, exosomal miR-181a-5p could inhibit hair follicle stem cell apoptosis but promote hair follicle stem cell proliferation in the angora rabbits [29]. MiR-149-5p could induce hair follicle stem cell differentiation in goats by promoting the expression of β-catenin [30]. In addition, many studies also revealed the regulation relationship of miRNA-lncRNA-mRNA in hair follicle development [31–33]. APA is a kind of post-transcriptional process that could change the binding site of miRNA and mRNA [34]. In this study, we found five protein-coding genes with more than 10 APA sites, including *ENSBGRG00000003029*, *CLIC4*, *COL1A2*, *CANX*, and *THRSP*. Chloride intracellular channel4 (CLIC4) has been reported to function in skin wound healing and *CLIC4* could upregulate TGF-β signaling in keratinocytes [35]. In addition, knockdown of the expression of *CLIC4* inhibited the expression of keratin 1 and keratin 10 which was induced by Ca^2+^ [36]. As we know keratin proteins are the main components of wool and hair shafts. Collagen Type I Alpha 2 Chain (COL1A2) is a member of the collagen family, previous studies revealed that *COL1A2* is one of the candidate genes related to cashmere production in goats [37]. Furthermore, Zhou revealed that *COL1A2* may also function in androgenetic alopecia [38]. Moreover, *COL1A2* also was the differently expressed gene in this study. Hence, we speculated that the APA event may also mediate the expression of *CLIC4* and *COL1A2* in hair growth.

The expression profiles could reflect the physical and biological processes of the organism. Functional enrichment analysis of DEIs revealed significant enrichment in pathways related to hair follicle and skin development. For example, the extracellular matrix-mediated crosstalk of multiple signaling pathways has been shown to regulate the density of hair follicles in sheep and wool bending in goats [37, 39]. It has been reported that enhanced focal adhesion can decrease cell migration in focal adhesion kinase-deficient mice [40], and the transition stage from telogen to anagen is associated with focal adhesion [41]. Li used RNA-seq and also revealed that ECM-receptor interaction and Focal adhesion were found in the hair regeneration stage of goats. Additionally, focal adhesion in the maintenance of hair follicle stem cell quiescence [42], and the hair follicle stem cell proliferation [43]. The PI3K-Akt signaling pathway has been extensively studied for its role in hair growth and skin development, including hair follicle regeneration [44], , hair follicle stem cell proliferation and differentiation [45], and hair-inducing dermal papilla cells [46]. Furthermore, we found some hair length-related genes, hair follicle cycle-related genes, and hair follicle growth-related genes were differently expressed between LHY and NHY. Consistent with the previous study of short-hair and long-hair rabbits [19], in this study, some keratin protein family genes and collagen family genes were also found with differential expression levels. Moreover, some keratin protein genes and collagen protein genes have also been found with differential expression levels in different stages of the hair cycle. Enhanced BMP signaling maintained the hair follicle stem cell’s quiescence and arrested hair follicle cell regeneration [47]. In this study, we found that *BMP4* was upregulated in the forehead of LHY, and *BMP1* was downregulated in the shoulder of LHY. The results indicated that the differential expression of BMP signaling may influence the biological process of hair follicles in the early anagen phase. In addition, *ARHGAP10* was also found upregulated in the shoulder of LHY. The study of X-Linked congenital hypertrichosis syndrome revealed that the *ARHGAP10* gene with a fragment insertion in the Mexican family with the universal overgrowth of terminal hair [48]. Hence, we speculated that *ARHGAP10* may regulate the terminal hair growth of mammals. The findings from the study indicate that the integration of Iso-seq and RNA-seq techniques is a valuable approach to analyze transcript profiles in yak skin. and the function of DEIs between LHY and NHY indicated that the hair follicle development and cycle affected the hair length growth difference.

## Conclusions

In this study, we found that the forehead and shoulder hair length of LHY were commonly longer than that of NHY, and the forehead hair length was significantly correlated to the shoulder hair length in the Tianzhu white yak. Besides, a total of 8849 potential novel loci, 66,989 isoforms 2,559 lncRNA, 239,734 AS events, and APA events of 2,760 genes were identified from Iso-seq data. The DEIs between LHY and NHY were analyzed by integrating Iso-seq and RNA-seq results. We found that some hair follicle development-related genes, such as *BMP4*, *KRT2*, *IGF2R*, and *COL1A2* in the forehead skin and *BMP1*, *KRT1*, *FGF5*, *COL1A2*, and *IGFBP5* in the shoulder group. Pathways enrichment analysis revealed that DEIs of the forehead and shoulder both significantly participated in hair growth-related signaling pathways, like ECM-receptor interaction, focal adhesion, and PI3K-Akt signaling pathway which indicated the role of DEIs in hair growth regulation. Furthermore, the DEIs-related protein interaction analysis suggested *COL1A2* and *COL3A1* could as the candidates for the hair growth in length difference. The findings comprehensively revealed the transcriptome complexity and identified some candidate transcripts in yak hair length. Overall, this study enhanced our understanding of the molecular mechanisms underlying the variation in hair length growth and hair follicle development in Tianzhu white yak.

## Materials and methods

### Hair length measurement and animal samples collection

As the temperature rises, the hair coat of Tianzhu white yak will be cut off annually in June. The length of hair at this time is the longest of the year. To compare the difference of hair length between LHY and NHY, we measured the hair length of 19 female yaks aged 3–5 years grazing in Tianzhu County, Gansu Province, China, including 10 long-haired yaks (LHY) and 9 normal-haired yaks (NHY). We used a ruler to measure the hair length of the yak at the forehead, shoulder, flank (skirt-hair), and tail. A bunch of hairs from a diameter of about 2 cm skin as a unit for hair length measurement and the shoulder hair length was measured 3 times per yak. The statistical difference in hair length between LHY and NHY was measured by the t-test method and performed by the SPSS software. Additionally, the hair length correlation analyses between the forehead and the shoulder were performed by R (cor (method = Pearson)). The data of hair length correlation analyses from 102 yaks including LHY and NHY. Then, we used the skin biopsy punches (8-mm-diameter) to collect skin samples of yak after injecting a local anesthetic (2% lidocaine) into the subcutaneous of the shoulder and forehead. A total of 16 skin samples were collected for the transcriptome study and the samples could be classified into four types contain skin from the forehead of LHY (LH), skin from the forehead of NHY (NH), skin from the shoulder of LHY (LS), and skin from the shoulder of NHY (NS). Detailed information on all experiment animals is listed in Additional file 1. All operations of sample collection were allowed by the herdsman. We had made all efforts to minimize the suffering of the animals studied here. The animal study is approved by The Animal Administration and Ethics Committee of Lanzhou Institute of Husbandry and Pharmaceutical Sciences of CAAS (Permit No. SYXK-2014-0002). A section of skin tissues was prepared for histological observation, and the rest samples were stored at − 80 °C for further study.

### RNA preparation

The total RNA of skin tissue was extracted using TRIzol reagent (Invitrogen, CA, USA) following the manufacturer’s protocol. RNA concentration and integrity were evaluated by the NanoDrop 2000 (NanoDrop Technologies, USA) and Agilent 2100 Bioanalyzer (Agilent, USA). Then, the quantified RNA samples were used for further cDNA library construction.

### Library construction and sequencing

A total of 16 RNA samples were equally mixed into a sample pool for the Iso-seq sequencing. The SMARTerTM PCR cDNA Synthesis Kit (Clonetech, USA) was used for the full-length cDNA synthesis. After PCR amplification, purification, and the cDNA fragments (< 1 kb) removal processes, the cDNA products were prepared for the SMRTbell library construction. Then, the purified and qualified library was performed on the PacBio Sequel II platform for third-generation sequencing. The RNA-seq libraries of 16 RNA samples were constructed using a VAHTS Universal V6 RNA-seq Library Prep Kit for MGI (Vazyme, Nanjing, China), and the raw data of RNA-seq was sequenced on the MGISEQ-2000 platform. The library construction and sequencing of Iso-seq and RNA-seq were performed by Frasergen Bioinformatics Co., Ltd. (Wuhan, China).

### Iso-Seq data processing and mapping

PacBio raw data were preprocessed using the SMRT Link, the workflow of polymerase reads analysis including low-quality reads and adapter removed, the circular consensus sequence (CCS) generation, and obtained the full-length non-concatemer sequence (FLNC) for the subsequent analysis. To avoid the SMRT sequencing error, high-quality short-read RNA-seq was used to correct the FLNC reads through LoRDEC v0.9 software (-k 21 -s 3) [49]. The quality control of RNA-seq raw data was conducted through SOAPnuke v2.1.0 (--lowQual 20, --nRate 0.005, --qualRate 0.5) [50]. The PID value represents the rate of the matched base to the reference sequence. Hence, we retained the FLNC with a high PID value. Then, the corrected FLNC sequence and uncorrected FLNC sequence were separately aligned to the Domestic yak reference genome (LU_Bosgru_v3.0) by using GMAP software (-e 1e-5 -k 10 –more-sensitive) [51]. Finally, the optimal reference mapping result was used for further study.

### Novel gene and isoforms identification

Based on the reference alignment result, the genome location of FLNC can assist in identifying the gene loci and isoform. To distinguish overlapping gene loci, the FLNC reads must meet certain criteria: they must align in the same direction, the overlap region in the alignment must be greater than 20%, and at least one exon overlap must be greater than 20%. For identifying isoforms of the same gene, the following rules apply: (1) the longer transcript is retained when FLNC transcripts have the same splicing sites; (2) 5’ degraded FLNC transcripts are removed; (3) an isoform must have at least two FLNC supports when the global PID is less than 99%; (4) and all splicing junctions must be supported by the reference genome annotation or RNA-seq alignment results when there is only one FLNC. Additionally, if the overlap between a transcript and a known gene is less than 20%, or if the transcript and known gene are transcribed in opposite directions, they are considered novel gene loci. A novel isoform of a known gene is identified if the sequenced transcript has a new splicing site or if one of the known isoforms or the sequenced transcript is not a single exon.

### Functional annotation of novel isoforms and LncRNA prediction

To better understand the function of identified novel isoforms, the sequence was aligned to five public databases, including NCBI non-redundant proteins (NR), Gene Ontology (GO), Cluster of Orthologous Groups of proteins (COG/KOG), KEGG Orthology (KO) and Swiss-Prot for the functional prediction by using Diamond v2.0.7 software (-e 1e-5 -k 10 -more-sensitive) [52]. After filtering the novel isoform with coding potential when blast against the NR, KOG, KEGG, and Swiss-Prot, lncRNA prediction was performed by using CNCI 2.0 (-m ve), CPC2 beta, CPAT 1.2.4, and PLEK 1.2 (-minlength 200). The overlapped result of four algorithms was considered as the final predicted lncRNA.

### AS and APA analysis

The post-transcriptional process is crucial for generating mature mRNA and transcript diversity, like alternative splicing (AS) events and alternative polyadenylation (APA). Pac Bio long-read sequencing is advantageous for identifying AS sites. AS events identification of the predicted isoform was analyzed using Astalavista v3.2 software (-t asta). The binding site altering of the poly (A) tail at the 3’ end of mRNA could affect the miRNA-mRNA binding region. APA of the isoform was identified using Tapis software [20], and the identified APA was confirmed by at least two FLNC sequences.

### Differential isoforms detection and functional analysis

PacBio sequencing provides more comprehensive transcript information. In this study, a novel reference annotation file was constructed by merging the known reference genome annotation file with PacBio novel isoforms annotation file. The expression differences of isoforms in yak skin tissue samples from LHY and NHY were investigated. Clean RNA-seq reads were aligned to the novel reference annotation using Bowtie2 v2.3.5 (-sensitive) [53], and the expression level of isoforms were quantified in fragments per kilobase million (FPKM) value using RSEM v1.3.3 [54]. FPKM values of isoforms were used to conduct the principal component analysis (PCA) to determine the consistency of the same sample group. And the DEIs detection was analyzed by DESeq2 (Fold change ≥ 2, and FDR ≤ 0.05) [55]. Then, the DEIs were enriched onto GO and KEGG databases by using the hypergeometric test for the functional analysis (*P-value* ≤ 0.05). The above analysis including the principal PCA analyses [56], DEIs detection [57], GO, and KEGG enrichment analyses [58] were conducted using OmicShare tool, a free online platform for data analysis.

### Protein-protein interaction network analyses

To investigate the key proteins of hair length growth of two compared groups. The DEIs-corresponding-proteins interaction relationship analyses were performed on the string (https://cn.string-db.org/), and the PPI network was constructed by the Cytoscape 3.7.2 software [59].

### The validation experiment of sequencing result

To validate the reliability of Iso-seq and RNA-seq results, a total of 6 known and novel isoforms, and six AS events were selected to validate by PCR method. The first strand cDNA for the PCR experiment was generated from 16 mixed RNA samples by using TaKaRa RNA PCRTM Kit (AMV) Ver.1.1 (Takara, Japan). The PCR reactions were per-formed using GoTaq® Green Master Mix, 2X (Promega). Besides, 15 differential isoforms were selected to validate the expression tendency between RNA-seq and qPCR. The cDNA for qPCR were respectively generated from 12 total RNA samples (3 individual samples in each group) by using PrimeScriptTM RT reagent Kit (Perfect Real Time) (Takara, Japan). The qPCR reaction was performed by using GoTaq® qPCR Master Mix (Promega, Madison, WI, USA). The relative expression level was calculated by the 2^−ΔΔCt^ method [60], and the *GAPDH* was chosen for the reference gene. All primers of validated genes were designed by the primer 5.0 software. Detailed information on validated genes and primer was supplied in Additional file 9 to file 11.

### Electronic supplementary material

Below is the link to the electronic supplementary material.

Supplementary Material 1

Supplementary Material 2

Supplementary Material 3

Supplementary Material 4

Supplementary Material 5

Supplementary Material 6

Supplementary Material 7

Supplementary Material 8

Supplementary Material 9

Supplementary Material 10

Supplementary Material 11

Supplementary Material 12

## Data Availability

The data supporting the conclusions of this study are available within the the manuscript or additional files. And the raw data of RNA-seq have been deposited in the Sequence Read Archive (SRA) of the National Center for Biotechnology Information (NCBI) database with the primary accession code PRJNA934110.

## Abbreviations

APA: Alternative polyadenylation
AS: Alternative splicing
CCS: Circular consensus sequence
DEIs: Differential isoforms
FLNC: Full-length non-concatemer sequence
FPKM: Fragments per kilobase million
Iso-seq: Isoform sequencing
LH: Skin from the forehead of long-haired yak
LHY: Long-haired yak
lncRNA: Long non-coding RNA
LS: Skin from the shoulder of long-haired yak
NH: Skin form the forehead of normal-haired yak
NHY: Normal-haired yak
NS: Skin from the shoulder of normal-haired yak
PPI: Protein-protein interaction
PCA: Principal component analysis
SMRT: Single Molecule Real-Time
