# Supplementary material for: Integrative analysis of Iso-Seq and RNA-seq data reveals transcriptome complexity and differential isoform in skin tissues of different hair length Yak
Source: BMC Genomics. 2024 May 21;25:498. doi: 10.1186/s12864-024-10345-8 (PMC11106907; doi:10.1186/s12864-024-10345-8)
Supplement: Supplementary file 2 — Supplementary Material 2 [file 12864_2024_10345_MOESM2_ESM.docx]

Table S1 Reads summary of the Iso-seq

| Type | Total number | Minimum length | Average length | Maximum length | N50 |
| --- | --- | --- | --- | --- | --- |
| Polymerase read | 1,188,555 | 51 | 90,023 | 463,662 | 168,272 |
| Subread | 60,550,561 | 51 | 1,675 | 294,658 | 2,284 |
| CCS | 841,259 | 59 | 2,297 | 13,133 | 2,669 |
| FLNC | 653,994 | 50 | 2,154 | 11,205 | 2,460 |
