# Supplementary material for: Integrative analysis of Iso-Seq and RNA-seq data reveals transcriptome complexity and differential isoform in skin tissues of different hair length Yak
Source: BMC Genomics. 2024 May 21;25:498. doi: 10.1186/s12864-024-10345-8 (PMC11106907; doi:10.1186/s12864-024-10345-8)
Supplement: Supplementary file 3 — Supplementary Material 3 [file 12864_2024_10345_MOESM3_ESM.docx]

Table S2 The mapping results of the pre-corrected and the post-corrected FLNC reads

| Category | Pre-correction | Post-correction | Merge |
| --- | --- | --- | --- |
| Unmapped | 6,720(1.03%) | 5,912(0.90%) | 5,899(0.90%) |
| Multiple best | 400(0.06%) | 401(0.06%) | 364(0.06%) |
| Low PID | 69,196(10.58%) | 64,903(9.92%) | 61,807(9.45%) |
| High quality map | 577,678(88.33%) | 582,778(89.11%) | 582,778(89.11%) |
