# Supplementary material for: Integrative analysis of Iso-Seq and RNA-seq data reveals transcriptome complexity and differential isoform in skin tissues of different hair length Yak
Source: BMC Genomics. 2024 May 21;25:498. doi: 10.1186/s12864-024-10345-8 (PMC11106907; doi:10.1186/s12864-024-10345-8)
Supplement: Supplementary file 4 — Supplementary Material 4 [file 12864_2024_10345_MOESM4_ESM.docx]

Table S3 Data summary of the loci and isoform annotated from ensemble and PacBio sequencing

| Feature | Annotation in Ensemble | Annotation in PacBio |
| --- | --- | --- |
| Total Loci | 22,689 | 21,285 |
| Loci <1K | 5,390(23.76%) | 2,245(10.55%) |
| Loci 1-2K | 8,618(37.98%) | 5,256(24.69%) |
| Loci 2-3K | 4,497(19.82%) | 5,016(23.57%) |
| Loci >=3K | 4,184(18.44%) | 8,768(41.19%) |
| Total isoform | 45,700 | 66,989 |
