# Supplementary figures and images for: Integrative analysis of Iso-Seq and RNA-seq data reveals transcriptome complexity and differential isoform in skin tissues of different hair length Yak
Source: BMC Genomics. 2024 May 21;25:498. doi: 10.1186/s12864-024-10345-8 (PMC11106907; doi:10.1186/s12864-024-10345-8)

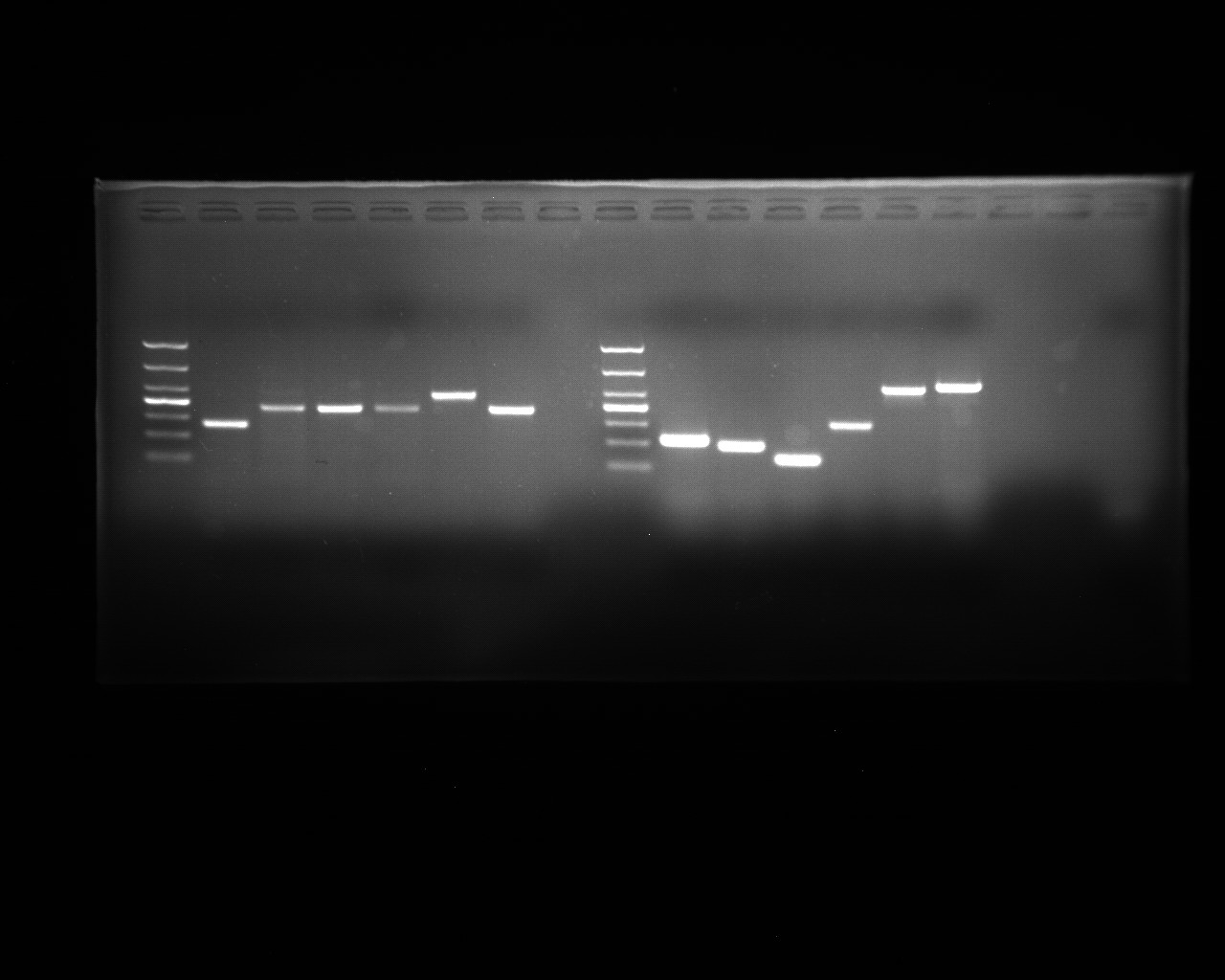

Supplement: Supplementary file 7 — Supplementary Material 7 [file 12864_2024_10345_MOESM7_ESM.jpg]

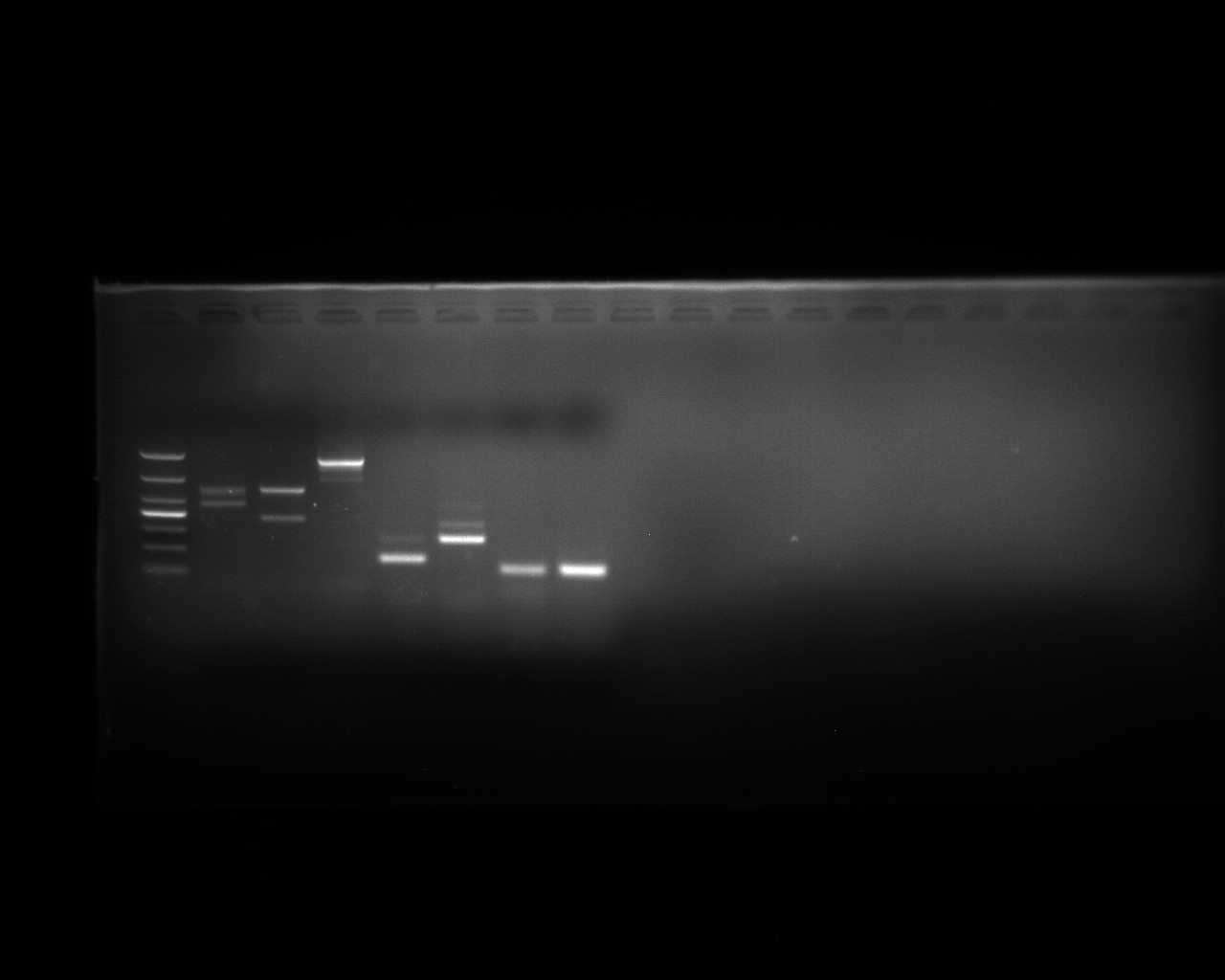

Supplement: Supplementary file 8 — Supplementary Material 8 [file 12864_2024_10345_MOESM8_ESM.jpg]
