# Supplementary material for: Integrative analysis of Iso-Seq and RNA-seq data reveals transcriptome complexity and differential isoform in skin tissues of different hair length Yak
Source: BMC Genomics. 2024 May 21;25:498. doi: 10.1186/s12864-024-10345-8 (PMC11106907; doi:10.1186/s12864-024-10345-8)
Supplement: Supplementary file 9 — Supplementary Material 9 [file 12864_2024_10345_MOESM9_ESM.docx]

Table S4 The primer information of known and novel isoforms

| Isoform | Type | Primer sequence (5’-3’) | Product length |
| --- | --- | --- | --- |
| ENSBGRT00000018314 | Known | CGAAACGGGCAACGGAT | 222bp |
|  |  | AGCACGGGTCGCTTTGAG |  |
| ENSBGRT00000033763 | Known | AAGTGTTAGGACCCGAA | 200bp |
|  |  | TGTGCGTTTGCGAGAGC |  |
| ENSBGRT00000028832 | Known | TGCTGCGGATATGGGTA | 112bp |
|  |  | TGCCTTGGAAAGCGTCG |  |
| 19.963.1 | Novel | AGTCCCTTCCATCTACCC | 316bp |
|  |  | GCTGCTGTCCTTGCTG |  |
| 20.651.1 | Novel | TGTCACCAACACCCAAC | 218bp |
|  |  | TTGCTTAACCACACCCCC |  |
| 27.60.1 | Novel | GCCCATTTCTTTCATTC | 573bp |
|  |  | ACCGTCAACCCCCAAAG |  |
