# Supplementary material for: Integrative analysis of Iso-Seq and RNA-seq data reveals transcriptome complexity and differential isoform in skin tissues of different hair length Yak
Source: BMC Genomics. 2024 May 21;25:498. doi: 10.1186/s12864-024-10345-8 (PMC11106907; doi:10.1186/s12864-024-10345-8)
Supplement: Supplementary file 10 — Supplementary Material 10 [file 12864_2024_10345_MOESM10_ESM.docx]

Table S5 The genome location and primer information of validated AS events

| name | type | Isoform | Genome location | Primer sequence (5’-3’) | Product length |
| --- | --- | --- | --- | --- | --- |
| AS1 | AA | ENSBGRT00000014616 | 54937920-,54938043- | GTTGGCAGGCAGGTTT | 225bp |
|  |  | 24.585.2 |  | TTTGGCTGGACGGTTC | 391 bp |
| AS2 | AD | ENSBGRT00000037626 | 27896395^,27896620^ | ACTGCTACGGGACTCTAC | 593 bp |
|  |  | 24.477.1 |  | AACACCACTCCTGCGACT | 368 bp |
| AS3 | ES | ENSBGRT00000000103 | 27834266-27834210^ | TGCCAGGAAGTCTACCC | 914 bp |
|  |  | 1.18.9 |  | TTGTCAACGGCGTCAGG | 858 bp |
| AS4 | ES | ENSBGRT00000019772 | 120344530-120344604^ | AAGACGGCTTCGGGGAA | 158 bp |
|  |  | 3.1163.3 |  | CTGCTGGAACTCAAGGC | 256 bp |
| AS5 | IR | ENSBGRT00000012112 | 22273576^22273493- | CTTTGAGGACTTCTTGGAC | 339 bp |
|  |  | 17.57.1 |  | CTCGGAGAGGTTGATGGT | 216 bp |
