# Supplementary material for: Integrative analysis of Iso-Seq and RNA-seq data reveals transcriptome complexity and differential isoform in skin tissues of different hair length Yak
Source: BMC Genomics. 2024 May 21;25:498. doi: 10.1186/s12864-024-10345-8 (PMC11106907; doi:10.1186/s12864-024-10345-8)
Supplement: Supplementary file 11 — Supplementary Material 11 [file 12864_2024_10345_MOESM11_ESM.docx]

Table S6 The primer information of validated differential genes

| Gene name | Primer sequence (5’-3’) | Product length |
| --- | --- | --- |
| ENSBGRG00000018450 | AAGTGTTAGGACCCGAA | 200bp |
|  | TGTGCGTTTGCGAGAGC |  |
| ENSBGRG00000015704 | TGCTGCGGATATGGGTA | 112bp |
|  | TGCCTTGGAAAGCGTCG |  |
| CYB5A | AAGTGTACGATTTGACC | 121bp |
|  | CGAGCATCTGTAGAGTGT |  |
| KAP13 | AGGAGACCTGCTGTGAG | 145bp |
|  | AAGTTACTGGAACCGAA |  |
| 5.927 | CCTCTGGGCTCCGAAAA | 266bp |
|  | TGTGGTGGGGGTATCTG |  |
| ENSBGRG00000005548 | GGTCCTGATGGCAAAAC | 129bp |
|  | TCCAGCAGCACCTTTAG |  |
| COL1A2 | ACTTTGCTGCTCAGTTTG | 228bp |
|  | CAGGTCGTCCAGGTTTT |  |
| COL3A1 | TTCCTCCGACTTCTCTC | 127bp |
|  | CTTCATTTGACCCCATC |  |
| ADAMTS2 | TCTGGAGAATGTTTGCCG | 180bp |
|  | TCCTCGTGGTTCAGCGTG |  |
| CCDC80 | AGAGTGAAAAGCAAGTG | 135bp |
|  | GATTCTGAGTGAAGGGT |  |
| SMIM10L1 | CCGAGGGTTGGAGTGTAA | 110bp |
|  | GCTTTGTGCTATGATTGG |  |
| PMEL | GTGGCAACCTCTGAAGTC | 187bp |
|  | TGGTATTTGAACCCGCA |  |
| GPX3 | GAATGGGGAGAAAGAGC | 202bp |
|  | GACAGAGTTGACCGTGG |  |
| ENSBGRG00000021504 | CCTGCCCTGTGTTTTATG | 131bp |
|  | GCAATCCCAGAGTTTTCC |  |
| ENSBGRG00000012868 | TGAGAAAAACAGCCCCAA | 183bp |
|  | AGAAATGTCGGTGCCCAG |  |
| GAPDH | CGATGCCCCCATGTTTGTGA | 126bp |
|  | GACGGTGGTCATAAGTCCCT |  |
