# Supplementary material for: Integrative analysis of Iso-Seq and RNA-seq data reveals transcriptome complexity and differential isoform in skin tissues of different hair length Yak
Source: BMC Genomics. 2024 May 21;25:498. doi: 10.1186/s12864-024-10345-8 (PMC11106907; doi:10.1186/s12864-024-10345-8)
Supplement: Supplementary file 12 — Supplementary Material 12 [file 12864_2024_10345_MOESM12_ESM.docx]

**Additional Files**

Additional file 1: The detailed information of experiment animals and hair length

Additional file 2: Reads summary of the Iso-seq

Additional file 3: The mapping results of the pre-corrected and the post-corrected FLNC reads

Additional file 4: Data summary of the loci and isoform annotated from ensemble and PacBio sequencing

Additional file 5: Annotation file of known and novel isoforms merged

Additional file 6: APA information of 7,315 genes

Additional file 7: The original full-length gels picture of 6 known and novel isoforms validation

Additional file 8: The original full-length gels picture of 5 AS events validation

Additional file 9: The primer information of known and novel isoforms

Additional file 10: The genome location and primer information of validated AS events

Additional file 11: The primer information of validated differential genes
